# Pembrolizumab versus sintilimab in patients with advanced NSCLC: a retrospective multicenter study with propensity-score matching analysis

**DOI:** 10.3389/fonc.2024.1422039

**Published:** 2024-12-05

**Authors:** Zhengzheng Xia, Juan Hong, Xiangyang Yu, Yuhua Ran, Huali Xie, Ziyuan Zhou, Jing Zuo, Tujia Chen, Jun Meng, Jun Yang

**Affiliations:** ^1^ Department of Pharmacy, National Cancer Center/National Clinical Research Center for Cancer/Cancer Hospital & Shenzhen Hospital, Chinese Academy of Medical Sciences and Peking Union Medical College, Shenzhen, China; ^2^ Department of Thoracic Surgery, National Cancer Center/National Clinical Research Center for Cancer/Cancer Hospital & Shenzhen Hospital, Chinese Academy of Medical Sciences and Peking Union Medical College, Shenzhen, China; ^3^ State Key Laboratory of Toxicology and Medical Countermeasures, Beijing Key Laboratory of Neuropsychopharmacology, Beijing Institute of Pharmacology and Toxicology, Beijing, China; ^4^ Department of Pharmacy, Hongkong University Shenzhen Hospital, Shenzhen, China; ^5^ Department of Pharmacy, Boai Hospital of Zhongshan, Zhongshan, China; ^6^ Department of Pharmacy, National Cancer Center/National Clinical Research Center for Cancer/Cancer Hospital, Chinese Academy of Medical Sciences and Peking Union Medical College, Beijing, China

**Keywords:** non-small-cell lung cancer, programmed death 1 inhibitors, effectiveness, safety, propensity-score matching, pembrolizumab, sintilimab

## Abstract

**Background:**

Programmed cell death protein 1 (PD-1) inhibitors are commonly used worldwide for the management of non-small cell lung cancer (NSCLC). However, it remains unclear whether pembrolizumab and sintilimab, two of the most widely used PD-1 inhibitors in China, have significantly different effects on patients with NSCLC. A multicenter retrospective cohort study was designed and implemented using propensity-score matching (PSM) analysis to compare the effectiveness and safety profiles of pembrolizumab and sintilimab in patients with advanced NSCLC undergoing comprehensive therapy.

**Methods:**

A total of 225 patients who received comprehensive therapy including pembrolizumab (n = 127) or sintilimab (n = 98), from 1 January to 31 December 2020 and met the eligibility criteria were included. PSM analysis (1:1) was performed to balance potential baseline confounding factors. For both treatments, Kaplan–Meier analysis and Cox regression were used to compare 1-year progression-free survival (PFS), disease control rate (DCR), objective response rate (ORR), and rates of all adverse events (AEs).

**Results:**

PSM analysis resulted in 63 matched pairs of patients. After PSM, the median PFS was 8.68 months in the sintilimab group and 9.46 months in the pembrolizumab group. The 1-year PFS showed no significant difference between the pembrolizumab and sintilimab groups before and after PSM (*P* = 0.873 and *P* = 0.574, respectively). Moreover, within the matched cohort, the pembrolizumab group had an ORR of 30.2% and a DCR of 84.1%, whereas the sintilimab group exhibited an ORR of 41.3% and a DCR of 88.9%. There were no significant differences in the ORR and DCR between the two groups (*P* = 0.248 and *P* = 0.629, respectively). The incidence of grade 3 or 4 treatment-related AEs was significantly higher in the pembrolizumab group than that in the sintilimab group (42.9% vs. 33.3%, *P* = 0.043). Multivariable Cox proportional hazards regression analysis indicated that the lines of treatment and regimens significantly influenced the PFS of patients (*P <*0.05).

**Conclusions:**

This study demonstrated the similar effectiveness of sintilimab and pembrolizumab in the treatment of patients with advanced NSCLC, with sintilimab potentially displaying a superior clinical safety profile.

**Clinical trial registration:**

https://www.medicalresearch.org.cn/, identifier MR4423000113.

## 1 Introduction

Lung cancer ranks as the leading cause of cancer-related deaths both in China and worldwide in 2022, and currently, advanced NSCLC is considered an incurable disease associated with poor prognosis (1). In this patient population, promising results have emerged from therapy with monoclonal antibody immune checkpoint inhibitors, particularly programmed death 1 (PD-1) inhibitors. Evidence has shown that these agents have greatly improved survival of NSCLC patients without driver mutations by blocking the interaction of PD-1 with its ligands (PD-L1 and PD-L2), thereby helping reverse T-cell anergy, exhaustion, and apoptosis (2, 3). By the end of June 2022, eight products were approved by the U.S. Food and Drug Administration (FDA), while 13 products were approved by the National Medical Products Administration (NMPA) of China (4, 5). Among these immunotherapies, pembrolizumab and sintilimab are two of the most clinically used PD-1 inhibitors in patients with NSCLC in China. Pembrolizumab was initially launched in the U.S. in 2017 and became available in China on 26 July 2018, while sintilimab was approved and marketed in China six months later, on 27 December 2018 (6, 7).

Four large randomized controlled phase III clinical trials have concluded that the efficacy and safety of adding sintilimab or pembrolizumab to standard chemotherapy were superior to chemotherapy alone in untreated locally advanced or metastatic NSCLC patients without epidermal growth factor receptor (EGFR) or anaplastic lymphoma kinase (ALK) mutations. Specifically, the KEYNOTE-189 (NCT02578680) trial concluded that first-line pembrolizumab plus pemetrexed-platinum substantially improved overall survival (OS) and progression-free survival (PFS) in 410 patients with metastatic nonsquamous NSCLC, regardless of PD-L1 expression or the presence of liver or brain metastases (8). The ORIENT-11 (NCT03607539) trial demonstrated that the addition of sintilimab to pemetrexed-platinum chemotherapy improved both PFS and OS in 397 Chinese patients with untreated locally advanced or metastatic nonsquamous NSCLC (9). The KEYNOTE-407 (NCT02775435) trial showed that in 278 patients with previously untreated metastatic squamous NSCLC, the administration of pembrolizumab plus carboplatin, along with either paclitaxel or nab-paclitaxel, resulted in significantly improved OS compared to those receiving chemotherapy alone (HR=0.71; 95% CI 0.58–0.88) (10–12). Furthermore, the ORIENT-12 (NCT03629925) study also demonstrated the promising results of sintilimab combined with gemcitabine and platinum (GP) therapy in 543 Chinese patients with squamous NSCLC (13). Based on these large clinical trials, it is evident that combining pembrolizumab or sintilimab with chemotherapy has shown favorable results in patients with advanced or metastatic NSCLC.

Although several PD-1 inhibitors are available, prescribing them poses challenges. The selection of which PD-1 inhibitor to use for the same indication is typically based on a consensus between patients and clinicians, given the lack of head-to-head studies comparing these drugs. This decision is particularly challenging for patients with advanced NSCLC in China, as there are limited data comparing the effectiveness and safety of the most commonly used PD-1 inhibitors in this population. Exploring the association between different anti-PD-1 agents, such as pembrolizumab and sintilimab, and survival and response rates in these patients could offer valuable insights into whether different PD-1 inhibitors independently impact tumor outcomes. Therefore, the purpose of this study was to re-evaluate the effectiveness and clinical safety of pembrolizumab and sintilimab in the treatment of patients with NSCLC through a multicenter retrospective cohort study with PSM analysis, as evidenced by the objective response rate (ORR), disease control rate (DCR), 1-year PFS, and adverse event (AE) rate.

## 2 Materials and methods

### 2.1 Study design

This was a retrospective multicenter cohort study conducted at two teaching hospitals in China (National Cancer Center Cancer Hospital, Beijing and Shenzhen). The study protocol was approved by the local ethics review board (no. YW2022-15), and a waiver of written informed consent was granted due to the retrospective nature of the study. Oral consent was obtained from the patients or their families when information was collected via telephone. The study adhered to the STROBE criteria and was conducted in accordance with the Declaration of Helsinki (14).

For this retrospective study, we utilized a database to screen NSCLC patients admitted to our two hospitals from 1 January to 31 December 2020. The diagnosis and clinical stage of NSCLC were confirmed according to the Eighth Edition of TNM staging of lung cancer-specified staging (15). The eligibility criteria for this study were as follows: (I) age 18 to 90 years; (II) a histologically confirmed diagnosis of stage IIIB/C or IV NSCLC with a negative oncogenic driver; (III) treatment with at least two courses of pembrolizumab or sintilimab; (IV) an Eastern Cooperative Oncology Group physical status (ECOG-PS) score of 0–3, and (V) adequate hematological, biochemical, and organ function. The exclusion criteria of the study were as follows: (I) severe organ failure (pulmonary, heart, hepatic, or renal diseases); (II) a history of angina, myocardial infarction, or interstitial pneumonia; (III) currently diagnosed with autoimmune system diseases; (IV) a history of any cancer or currently diagnosed with cancers other than NSCLC; and (V) use of two or more different PD-1 inhibitors during treatment. The patients were divided into pembrolizumab and sintilimab groups based on the type of PD-1 inhibitor used. The patients received pembrolizumab or sintilimab intravenously at a fixed dose of 200 mg every three weeks. All patients were re-examined using ultrasound or CT and chest X-ray at six weeks after the initial PD-1 inhibitor treatment and were then routinely followed-up at two-month intervals thereafter.

### 2.2 Data collection and assessment

The data for this study were collected from a database of NSCLC patients. All data collectors were blinded to the research aims during data abstraction. In each center, all data were input by two trained reviewers using Excel, and all records were reviewed by another independent investigator.

Demographic information, including sex and age at diagnosis, was also collected. Clinical characteristics of patients were also abstracted, including ECOG performance status, histological type, pathological stage, brain metastasis status, lines of treatment, PD-L1 level, comorbidities, smoking status, and treatment regimens. Comorbidities included heart failure, hypertension, diabetes mellitus, chronic kidney disease, chronic lung disease, and cerebrovascular disease. Effectiveness evaluation of the study included ORR, DCR, and PFS. PFS was defined as the time from the date of first checkpoint inhibitor administration until disease progression or death, whichever occurred first. PFS assessments were performed using chest contrast-enhanced CT or chest radiography, abdominal ultrasound or abdominal CT, repeat brain MRI, and bone scans until radiological disease progression or death.

Tumor remission was defined as a reduction in the size of measurable lesions by at least 30%, with no new lesions appearing, whereas tumor progression was defined as the new appearance of intra- or extra-thoracic tumor nodules according to the *Response Evaluation Criteria in Solid Tumors (RECIST 1.1 Version)* (16). All adverse events and severe adverse events were recorded and evaluated according to the *Cancer Institute Common Terminology Criteria for Adverse Events (CTCAE Version 5.0)* (17). Patients who were still receiving immunotherapy at the data cutoff were censored. Patients who underwent at least one follow-up imaging assessment were evaluated for radiological response and time-to-progression. Patients who were still alive and without radiologically confirmed progression were censored at the date of the last contact or data cut-off. The follow-up period ended on 31 March 2022, and the data were censored for patients who were alive as of that date.

### 2.3 Sample size and power

Based on published studies (8–13), we assumed that the median time to progression of NSCLC would be approximately 8.9 months in the sintilimab group and 9.0 months in the pembrolizumab group. To achieve a two-sided α level of 0.05 and 90% power, approximately 255 patients would be required. Considering the potential loss to follow-up, a total of 126 patients (63 patients per group) would be required, assuming a loss rate of 10%.

### 2.4 Statistical analysis

Categorical variables are reported as number (n) or proportion (%), while continuous variables are expressed as mean ± standard deviation (SD) or median [25% interquartile range, 75% interquartile range]. Student’s t-test or Mann–Whitney U test was used to compare continuous variables between the two treatment groups. Categorical variables were compared using the χ^2^ test with the Yates correction or Fisher’s exact test (when total sample size was <40 or expected frequency was <1). Univariate and multivariable Cox regression analyses were performed to identify the independent predictive factors of prognosis. The PFS rates were compared between the pembrolizumab and sintilimab groups using Kaplan–Meier curves generated by the log-rank test with a two-sided significance level of 5%. Multivariable Cox proportional hazards regression analysis was performed to adjust for covariates associated with PFS. Multivariable regression curves were generated after the multivariable Cox regression analysis.

PSM analysis, as described previously (16), was used to eliminate imbalances in the baseline characteristics between the two groups. We applied 1:1 nearest-neighbor matching without replacement to ensure that the conditional bias was minimized. For each patient receiving sintilimab, a patient receiving pembrolizumab with a minimum distance propensity score was matched. To determine the most appropriate caliper widths, caliper widths of 0.05, 0.02, and 0.001 were used for PSM. Finally, a caliper width of 0.02 met the criteria for both preferable homogeneity and minor loss of sample size.

All statistical tests were two-sided, and statistical significance was set at *p*-values <0.05. Statistical and PSM analyses were performed using IBM SPSS Statistics (version 26.0; SPSS Inc., Armonk, NY, USA).

## 3 Results

### 3.1 The baseline characteristics of patients before and after PSM

**Figure 1:**
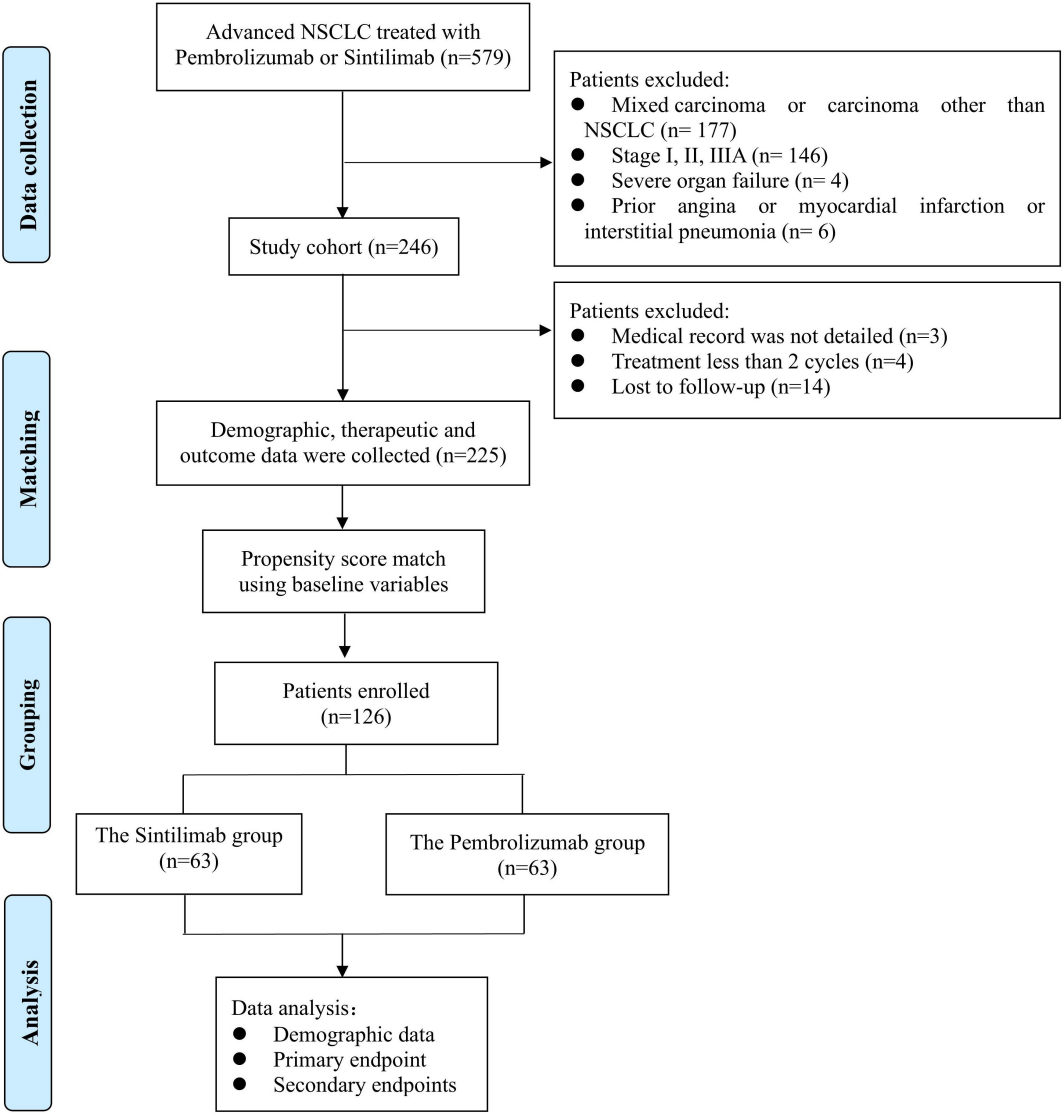
The flow chart of screened patients.

From 1 January to 31 December 2020, 579 patients with advanced NSCLC who presented with pembrolizumab or sintilimab at two hospitals were screened. Among these, 246 met the eligibility criteria and were enrolled in the study. Of these, 14 patients were lost to follow-up, four patients were treated with ICIs for less than two cycles, and three patients had incomplete medical records (**Figure 1**). Ultimately, 127 and 98 patients remained in the sintilimab and pembrolizumab groups, respectively (**Table 1**).

**Table 1:** Comparison of demographic and clinical characteristics of the patients enrolled in the study before and after propensity-score matching.

Variables	Before PSM			After PSM		
	Sintilimab group (n = 98)	Pembrolizumab group (n = 127)	*p*-value	Sintilimab group (n = 63)	Pembrolizumab group (n = 63)	*p*-value
Gender			0.093			0.607
Male, n (%)	83 (84.7%)	96 (75.6%)		53 (83.1%)	50 (79.4%)	
Female, n (%)	15 (15.3%)	31 (24.4%)		10 (15.9%)	13 (20.6%)	
Age,			0.865			0.613
mean ± SD (years)	61.1 ± 9.8	60.9 ± 10.2		60.6 ± 9.9	61.9 ± 10.7	
≤60, n (%)	44 (44.9%)	53 (41.7%)		30 (47.6%)	25 (39.7%)	
>60, n (%)	54 (55.1%)	74 (58.3%)		33 (52.4%)	38 (60.3%)	
PS score, n (%)			0.079			0.561
0	0 (0)	0 (0)		0 (0)	0 (0)	
1	53 (54.1%)	69 (54.3%)		33 (52.4%)	30 (47.6%)	
2	19 (19.4%)	17 (13.4%)		28 (44.4%)	30 (47.6%)	
3	26 (26.5%)	41 (32.3%)		2 (3.1%)	3 (4.8%)	
Histological type, n (%)			0.754			0.690
Adenocarcinoma	66 (67.3%)	83 (65.4%)		39 (61.9%)	42 (66.7%)	
Squamous carcinoma	32 (32.7%)	44 (36.6%)		24 (38.1%)	21 (33.3%)	
TNM stage, n (%)			0.325			0.826
IIIA	6 (6.1%)	6 (4.7%)		4 (6.3%)	6 (9.5%)	
IIIB	16 (16.3%)	11 (8.7%)		10 (15.9%)	7 (11.1%)	
IIIC	1 (1.0%)	7 (5.5%)		1 (1.6%)	4 (6.3%)	
IV	75 (76.5)	103 (81.1%)		48 (76.2%)	46 (73.0%)	
Bain metastases, n (%)			0.121			0.607
Yes	16 (16.3%)	12 (9.4%)		7 (11.1%)	10 (15.9%)	
No	82 (83.7%)	115 (90.6%)		56 (88.9%)	53 (84.1%)	
Lines, n (%)			0.726			0.804
First line	51 (52.0%)	69 (54.3%)		34 (54.0%)	36 (57.1%)	
Second line	19 (19.4%)	17 (13.4%)		14 (22.2%)	7 (11.1%)	
Third or more line	28 (28.6%)	41 (32.3%)		15 (23.8%)	20 (31.7%)	
PD-L1 level, n (%)			0.000			0.435
Positive	81 (82.7%)	53 (41.7%)		13 (20.6%)	24 (38.1%)	
Negative	17 (17.3%)	74 (58.3%)		50 (79.4%)	39 (61.9%)	
Comorbidities, n (%)			0.355			0.557
Yes	41 (41.8%)	61 (48.0%)		28 (44.4%)	32 (50.8%)	
No	57 (58.2%)	66 (52.0%)		35 (55.6%)	31 (49.2%)	
Smoking status, n (%)			0.297			0.597
Never	43 (43.9%)	47 (37.0%)		22 (34.9%)	26 (41.3%)	
Former or current	55 (56.1%)	80 (63.0%)		41 (65.1%)	37 (58.7%)	
Regimen, n (%)			0.079			0.261
with radiotherapy	44 (44.9%)	72 (56.7%)		29 (46.0%)	31 (49.2%)	
without radiotherapy	54 (55.1%)	55 (43.3%)		34 (54.0%)	32 (50.8%)	

The baseline patient characteristics are shown in **Table 1**. Before PSM analysis, female patients (24.4% vs. 15.3%, *P* = 0.093), patients with a performance status (PS) score of three (32.3% vs. 26.5%, *P* = 0.079), patients with negative PD-L1 expression (58.3% vs. 17.3%, *P* = 0.000), and patients receiving chemoradiotherapy combination regimens (56.7% vs. 44.9%, *P* = 0.079) were more prevalent in the pembrolizumab group than in the sintilimab group, although these differences were not statistically significant (**Table 1**). After matching in a 1:1 ratio, 63 paired patients were included in the analysis. The baseline characteristics of the patients were examined, revealing a comparable balance in all matched characteristics between the sintilimab and pembrolizumab groups.

### 3.2 The comparison of the clinical outcomes between two groups

**Figure 2:**
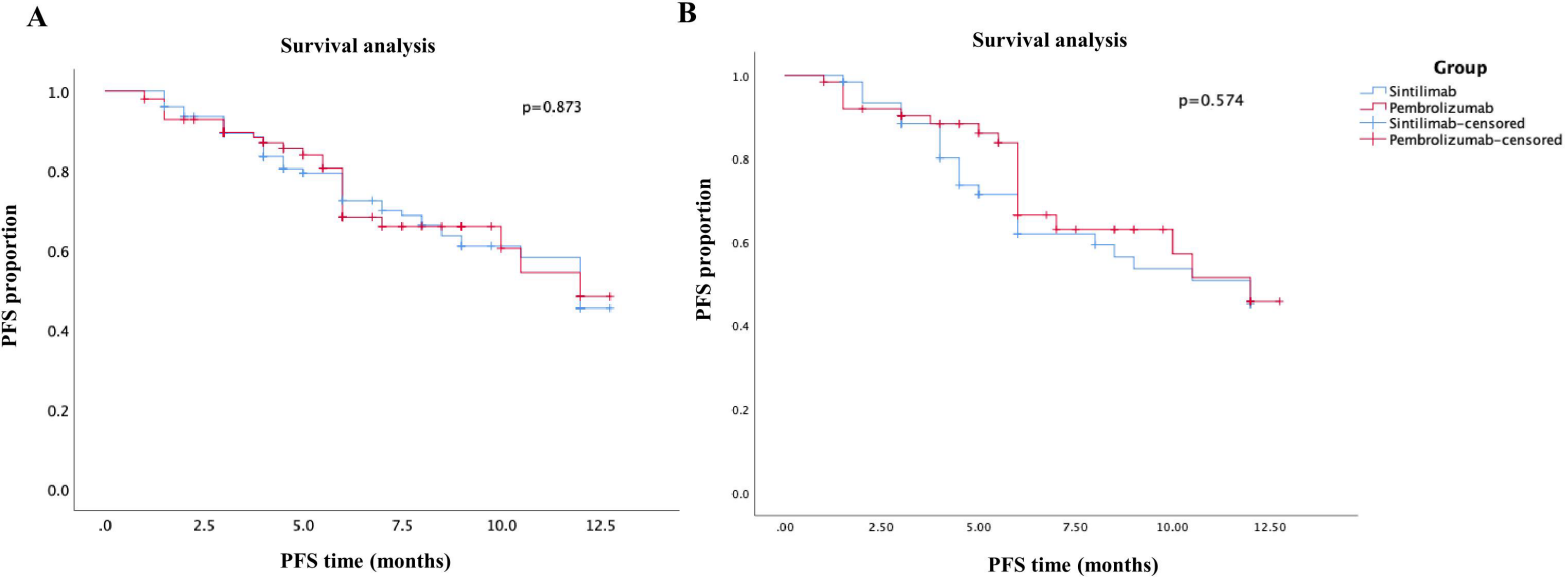
Kaplan–Meier curves of PFS of patients in the sintilimab group and pembrolizumab group. **(A)** before PSM analysis; **(B)** after PSM analysis.

The primary outcome of the study was 1-year PFS, and the secondary outcomes included ORR, DCR after 6 months, and clinical safety. After PSM analysis, the median PFS was 8.68 months in sintilimab group compared to 9.46 months in pembrolizumab group (**Figure 2**). Moreover, there was no significant difference in the 1-year PFS between the pembrolizumab and sintilimab groups before and after PSM (*P* = 0.873 and *P* = 0.574, respectively) (**Figure 2**). The secondary outcomes of the two groups are presented in **Table 2**. After PSM, the ORR was 30.2% in the pembrolizumab group and 41.3% in the sintilimab group, whereas the DCR was 84.1% and 88.9%, respectively. We found no significant difference in the ORR and DCR between the two groups (*P* = 0.248 and *P* = 0.629, respectively) (**Table 2**). Additionally, the incidence of grade 3 or 4 treatment-related adverse events (TRAEs) was significantly higher in the pembrolizumab group than in the sintilimab group (42.9% vs. 33.3%, *P* = 0.043) (**Table 2**). These results suggest that with similar effectiveness of PD-1 inhibitors between the two groups, the administration of sintilimab in NSCLC patients may exhibit superior clinical safety compared to pembrolizumab.

**Table 2:** The clinical outcomes of the sintilimab and pembrolizumab groups.

Outcomes	Before PSM (n = 225)			After PSM (n = 126)		
	Pembrolizumab group (n = 127)	Sintilimab group (n = 98)	*P-*Value	Pembrolizumab group (n = 63)	Sintilimab group (n = 63)	*P*-Value
ORR	47 (37.0%)	35 (35.7%)	0.842	19 (30.2%)	26 (41.3%)	0.248
DCR	111 (87.4%)	85 (86.7%)	0.882	51 (84.1%)	56 (88.9%)	0.629
3–4 grade TRAEs	61 (48.0%)	41 (41.8%)	0.664	27 (42.9%)	21 (33.3%)	0.043

### 3.3 Univariate and multivariate Cox-regression analyses for progression-free survival

First, Kaplan–Meier analysis or a univariable Cox proportional-hazard model was utilized to screen for variables that significantly influenced PFS. As shown in **Supplementary Table S1**, after PSM analysis, sex, brain metastasis status, lines of treatment, and treatment regimens significantly influenced patient PFS (P <0.05). The significant variables listed in **Supplementary Table S1** were entered into multivariate Cox regression model analysis. Again, after PSM analysis, lines of treatment, and treatment regimens significantly affected patient PFS (P <0.05) (**Table 3**).

**Table 3:** Multivariable Cox-regression model analysis of PFS.

Independent predictive factor	Before PSM			After PSM		
	HR	95% CI	*p*-value	HR	95% CI	*p*-value
Pembrolizumab vs. Sintilimab	0.815	0.482–1.379	0.446	0.859	0.466–1.583	0.627
Male vs. Female	0.696	0.362–1.338	0.277	0.862	0.368–3.871	0.733
Age (>60 yr vs. ≤60 yr)	0.556	0.350–0.884	0.695	0.721	0.390–1.331	0.295
PS score (≥2 vs. 0–1)	0.576	0.174–1.909	0.367	1.540	0.796–2.978	0.200
Histological type (Squamous carcinoma vs. Adenocarcinoma)	1.016	0.597–1.728	0.954	1.074	0.494–2.336	0.857
TNM stage (IV vs. III)	0.880	0.465–1.665	0.695	1.206	0.376–3.871	0.753
Bain metastases (Yes vs. No)	1.168	0.897–3.171	0.105	1.816	0.859–3.839	1.118
Lines (First line vs. Second or more line)	2.403	1.464–3.945	0.001	2.865	1.438–5.704	0.03
Comorbidity (Yes vs. No)	1.588	0.995–2.534	0.052	1.103	0.585–2.336	0.761
Smoking status (No vs. Yes)	0.859	0.476–1.549	0.612	0.699	0.316–1.546	0.376
Regimen (Without radiotherapy vs. With radiotherapy)	0.492	0.291–0.799	0.005	0.314	0.149–0.662	0.002

## 4 Discussion

Although our study was a retrospective evaluation, it presented a comparison between these two agents in patients with advanced NSCLC, contributing to the ongoing exploration in this area. Well-matched cohorts of patients were established using PSM analysis to compare the clinical outcomes. Our findings revealed no significant differences in the 1-year PFS, DCR, and ORR in patients with advanced NSCLC undergoing sintilimab or pembrolizumab treatment. Furthermore, the sintilimab group exhibited a significantly lower incidence of grade 3 or 4 treatment-TRAEs compared to the pembrolizumab group.

In our study, the efficacy and safety of pembrolizumab and sintilimab were similar to those reported in four large clinical randomized controlled phase III trials. In patients with adenocarcinoma, the median PFS were 9.2 months and 9.0 months, respectively, in the pembrolizumab arm of the KEYNOTE-189 study and in the sintilimab arm of the ORIENT-11 study (8, 11). Additionally, the incidence of grade 3–4 AEs was 71.9% in the pembrolizumab combination arm for patients with squamous carcinoma. The median PFS was 8.0 months and 6.7 months, respectively, in the pembrolizumab arm of the KEYNOTE-407 study and in the sintilimab arm of the ORIENT-12 study (9, 13). Furthermore, in the KEYNOTE-407 study, grade 3 to 4 AEs occurred in 74.1% of the patients receiving pembrolizumab plus chemotherapy.

The influence of different immune checkpoint inhibitors (ICIs) on the outcomes of patients with different types of tumors has been a topic of interest in recent years. Several studies have compared the efficacy and toxicity spectra of different ICIs using meta-analysis methods (18–20). However, the conclusions of these studies tend to vary, and evidence from head-to-head comparisons of different ICIs is lacking. To investigate how different ICIs are administered to different groups of patients with advanced NSCLC, other indirect comparisons or analytical approaches have been adopted. Nagasaka et al. conducted a retrospective study using the US Flatiron Health electronic health record-derived deidentified database to assess the generalizability of ORIENT-11 trial results to a real-world patient cohort with advanced NSCLC in the US (21). After adjusting the inverse probability weights between ORIENT-11 patient data and US patient data, PFS remained superior for the sintilimab plus chemotherapy group, and safety outcomes were consistent. Considering that sintilimab has a lower cost (approximately 24,000 USD per year) than pembrolizumab (approximately 87,000 USD per year), it provides an innovative and feasible treatment option for locally advanced or metastatic NSCLC that may not have access to these high-priced immunotherapy agents (22).

This study has several limitations. First, this was a retrospective analysis that utilized PSM to reduce bias in patient selection. Although PSM was employed to minimize the impact of the observed confounders, several unobserved factors may have influenced the outcomes. Key potential confounders that were not accounted for included baseline health status, which encompasses patients’ pretreatment comorbidities that could affect their tolerance and survival outcomes. Additionally, variations in immune system function, particularly baseline immune status and relevant biomarkers of immune response, may influence the efficacy of immune checkpoint inhibitors; however, data on these immune profiles were not included in the analysis. Lifestyle factors, such as smoking, alcohol consumption, diet, and exercise, also remain unmeasured, potentially confounding the treatment-outcome relationship. Finally, critical clinical parameters such as performance status and previous therapies were not fully captured, further contributing to potential biases in the findings. Addressing these limitations in future studies will enhance the robustness of the results.

Second, distinguishing immune-related adverse events (irAEs) from common AEs is challenging (23, 24). The different types of AEs also influenced the summary of the safety profiles of PD-1 inhibitors. Third, our study involved only Chinese population at two cancer centers. Fourth, the number of patients treated with pembrolizumab was larger than that of patients treated with sintilimab. This difference can be attributed to the timing of market availability. Pembrolizumab was first launched in the US in 2017 and was subsequently marketed in China on 26 July 2018, whereas sintilimab was launched in China on 27 December 2018.

In the future, extending the follow-up period to gather long-term survival data and assessing treatment response sustainability will be essential. Comparative research on the cost-effectiveness of pembrolizumab and sintilimab in real-world clinical settings can guide healthcare resource allocation. Additionally, conducting head-to-head comparisons with new PD-1 inhibitors entering the market will offer critical insights for selecting optimal immunotherapy agents for patients with advanced NSCLC.

## 5 Conclusions

In summary, sintilimab has shown effectiveness comparable to pembrolizumab in real-world patients with advanced NSCLC, and sintilimab may have a more favorable clinical safety profile.

## Data Availability

The raw data supporting the conclusions of this article will be made available by the authors, without undue reservation.
