# Supplementary material for: Pembrolizumab versus sintilimab in patients with advanced NSCLC: a retrospective multicenter study with propensity-score matching analysis
Source: Front Oncol. 2024 Dec 5;14:1422039. doi: 10.3389/fonc.2024.1422039 (PMC11655332; doi:10.3389/fonc.2024.1422039)
Supplement: Supplementary file 1 [file Supplementaryfile1.docx]

Supplementary Material

**Supplementary Table S1 Results of univariable Cox-regression model analysis on PFS**

| **Variables** | **Before PSM** | | | **After PSM** | | |
| --- | --- | --- | --- | --- | --- | --- |
|  | **HR** | **95% CI** | ***p* value** | **HR** | **95% CI** | ***p* value** |
| Pembrolizumab vs. Sintilimab | 1.039 | 0.643-1.676 | 0.877 | 1.179 | 0.652-2.133 | 0.586 |
| Male vs. Female | 0.633 | 0.379-1.056 | 0.080 | 0.518 | 0.271-0.990 | 0.046 |
| Age (>60 yr vs. ≤60 yr) | 0.547 | 0.348-0.862 | 0.009 | 0.565 | 0.315-1.016 | 0.056 |
| PS score (≥2 vs. 0-1) | 1.339 | 0.652-2.012 | 0.002 | 1.048 | 0.578-1.901 | 0.877 |
| Histological type  (Squamous carcinoma vs. Adenocarcinoma) | 0.952 | 0.583-1.555 | 0.845 | 1.665 | 0.824-3.366 | 0.156 |
| TNM stage  (IV vs. III) | 1.061 | 0.582-1.933 | 0.846 | 2.454 | 0.876-6.875 | 0.088 |
| Bain metastases  (Yes vs. No) | 2.068 | 1.155-3.702 | 0.014 | 2.612 | 1.340-5.091 | 0.005 |
| Lines  (First line vs. Second or more line) | 2.022 | 1.276-3.203 | 0.002 | 2.239 | 1.225-4.093 | 0.009 |
| Comorbidity (Yes vs. No) | 1.420 | 0.904-2.231 | 0.128 | 1.012 | 0.564-1.818 | 0.967 |
| Regimen  (Without radiotherapy vs. With radiotherapy) | 0.636 | 0.403-1.004 | 0.052 | 0.384 | 0.197-0.748 | 0.005 |
