# Supplementary material for: Pembrolizumab versus sintilimab in patients with advanced NSCLC: a retrospective multicenter study with propensity-score matching analysis
Source: Front Oncol. 2024 Dec 5;14:1422039. doi: 10.3389/fonc.2024.1422039 (PMC11655332; doi:10.3389/fonc.2024.1422039)
Supplement: Supplementary file 2 [file Supplementaryfile2.docx]

**Supplementary Table S2 3-4 grade TRAEs of Pembrolizumab group and Sintilimab group**

| \| 3-4 grade TRAEs \| Pembrolizumab group  (n=63) \| Sintilimab group  (n=63) \| \| --- \| --- \| --- \| \|  \| \| Any terms \| 27(42.9%) \| 21(33.3%) \|  \| \| Anemia \| 9(14.3%) \| 8(12.7%) \|  \| \| Neutropenia \| 10(15.9%) \| 8(12.7%) \|  \| \| Thrombocytopenia \| 4(6.3%) \| 3(4.8%) \|  \| \| White blood cell count decreased \| 8(12.7%) \| 6(9.5%) \|  \| \| Nausea \| 2(3.2%) \| 2(3.2%) \|  \| \| Vomiting \| 1(1.6%) \| 1(1.6%) \|  \| \| Constipation \| 2(3.2%) \| 2(3.2%) \|  \| \| Diarrhea \| 2(3.2%) \| 1(1.6%) \|  \| \| Transaminases increased \| 4(6.3%) \| 3(4.8%) \|  \| \| Cough \| 4(6.3%) \| 4(6.3%) \|  \| \| Weight decreased \| 2(3.2%) \| 1(1.6%) \|  \| \| Rash \| 1(1.6%) \| 1(1.6%) \|  \| \| Immune-related AEs \| 7(11.1%) \| 5(7.9%) \|  \| |
| --- | --- | --- | --- | --- | --- | --- | --- | --- | --- | --- | --- | --- | --- | --- | --- | --- | --- | --- | --- | --- | --- | --- | --- | --- | --- | --- | --- | --- | --- | --- | --- | --- | --- | --- | --- | --- | --- | --- | --- | --- | --- | --- | --- | --- | --- | --- | --- | --- | --- | --- | --- | --- | --- | --- | --- | --- | --- | --- | --- | --- |
|  |
| TRAEs: treatment-related adverse events; AEs: Adverse Events. |
|  |
|  |
|  |
|  |
|  |
|  |
|  |
|  |
|  |
|  |
|  |
|  |
|  |
